# Analysis of Stored mRNA Degradation in Acceleratedly Aged Seeds of Wheat and Canola in Comparison to Arabidopsis

**DOI:** 10.3390/plants9121707

**Published:** 2020-12-04

**Authors:** Liang Zhao, Hong Wang, Yong-Bi Fu

**Affiliations:** 1 Department of Biochemistry, Microbiology and Immunology, University of Saskatchewan, 107 Wiggins Rd, Saskatoon, SK S7N 5E5, Canada; liz704@usask.ca; 2 Plant Gene Resources of Canada, Saskatoon Research and Development Centre, Agriculture and Agri-Food Canada, 107 Science Place, Saskatoon, SK S7N 0X2, Canada

**Keywords:** *β* value, canola, real-time quantitative PCR (qPCR), seed aging, seed germination, seed longevity, stored mRNA degradation, wheat

## Abstract

Seed aging has become a topic of renewed interest but its mechanism remains poorly understood. Our recent analysis of stored mRNA degradation in aged Arabidopsis seeds found that the stored mRNA degradation rates (estimated as the frequency of breakdown per nucleotide per day or *β* value) were constant over aging time under stable conditions. However, little is known about the generality of this finding to other plant species. We expanded the analysis to aged seeds of wheat (*Triticum aestivum*) and canola (*Brassica napus*). It was found that wheat and canola seeds required much longer periods than Arabidopsis seeds to lose seed germination ability completely under the same aging conditions. As what had been observed for Arabidopsis, stored mRNA degradation (∆Ct value in qPCR) in wheat and canola seeds correlated linearly and tightly with seed aging time or mRNA fragment size, while the quality of total RNA showed little change during seed aging. The generated *β* values reflecting the rate of stored mRNA degradation in wheat or canola seeds were similar for different stored mRNAs assayed and constant over seed aging time. The overall *β* values for aged seeds of wheat and canola showed non-significant differences from that of Arabidopsis when aged under the same conditions. These results are significant, allowing for better understanding of controlled seed aging for different species at the molecular level and for exploring the potential of stored mRNAs as seed aging biomarkers.

## 1. Introduction

The last decade has seen an increased interest in studies of seed longevity [1,2,3,4,5,6,7,8,9,10,11,12,13,14]. This largely reflects the need to address the challenges faced in the efforts to manage and conserve ex situ seed germplasm worldwide [15]. There are currently more than 7.4 million accessions of seed germplasm conserved in 1750 genebanks around the world, and conserved seeds will eventually lose their ability to germinate after long-term storage [3,16,17]. Thus, it is critical to monitor seed aging for timely regeneration of conserved germplasm, but such seed aging monitoring is technically challenging. Seed germination testing is the recommended method to evaluate germination ability and it is widely used in genebank management [18]. However, seed germination tests are not always accurate at predicting seed longevity [16] or the lack of seed viability during the early stages of germination loss [19] and these tests hardly reflect the underlying mechanisms of seed aging [2,20]. Additionally, different species may have different expected seed longevity under the same storage conditions [8,16], making seed longevity prediction more difficult for genebank management. Thus, some studies have been conducted to explore seed aging processes and status with the hope of facilitating the development of effective biomarkers for monitoring seed aging under storage conditions [2,3,20,21,22,23,24,25,26,27,28,29]. The studies included the analyses of glutathione half-cell reduction potential [28], volatile organic compound fingerprint [25,26,27], RNA integrity [29,30], thermal profile [24], and molecular mobility [23] in aged seeds.

Seed aging or seed deterioration is commonly described as the loss of seed quality or viability over time [31]. It is a complex biological trait and involves a network of molecular, biochemical, physiological, and metabolic processes [4,6,7,32]. Many factors were suggested to play roles in seed aging, and reactive oxygen species (ROS) were considered as the major factor [33,34]. ROS accumulate in seeds and attack macromolecules continuously, resulting in damage and dysfunction of macromolecules [33]. It has been reported that wheat seed aging is associated with an increased level of peroxide, and seed viability has a good correlation with the level of ROS-scavenging molecules [35]. Other factors contributing to seed aging include the damage to macromolecules like proteins [36,37,38], nucleic acids (including DNA and RNA) [14,29,30,39,40,41], lipids [42,43], and organelles [44]. However, we are still far from understanding the causes of seed aging and death, particularly under long-term storage in a controlled environment [4,6].

Recently, seed-stored mRNAs have received some attention [14,29,30,41,45,46,47]. Stored mRNAs accumulate in seeds at the late stage of embryogenesis and could remain intact during long periods of ex situ genebanking, subsequently playing a role as a template in protein synthesis during seed germination [48,49,50]. Some stored mRNAs may also encode critical enzymes required for restarting cellular metabolism after seed rehydration and without them seed germination could be impaired [32,40,50,51,52]. In dry seeds, RNA bases can be modified or damaged by oxidative reagents during storage, and the most abundant base modification is 8-oxo-G under an oxidative environment [53,54]. In the germination process, the modified or damaged mRNA bases could negatively affect mRNA metabolism and protein translation, resulting in mRNA stalling on the ribosome and/or reduced/truncated protein production [55]. An early study showed a reduced ability to degrade stored mRNA and to *de novo* synthesize mRNA in severely aged wheat seeds and reported a correlation between seed germination percentage and the ability to synthesize mRNA *de novo* in wheat seeds [56].

As stored mRNAs have been suggested to have the potential to offer useful biomarkers for monitoring seed aging [14,29,30,41], we also conducted a study on seed-stored mRNAs and their associations with seed aging [45]. Using the model plant Arabidopsis (*Arabidopsis thaliana*), we showed that all stored mRNAs analyzed were gradually degraded in naturally and acceleratedly aged seeds and that the difference in Ct values from real-time quantitative PCR (qPCR) between aged and control seeds (or ∆Ct value) was highly correlated with the mRNA fragment size and seed aging time [45]. More interestingly, the frequency of the breakdown at one nucleotide level per day (or *β* value) for most assayed mRNAs remained fairly constant under the same aging conditions [45], suggesting that *β* value could be a unique and useful measurement in seed aging evaluation. However, the generality of these findings to the other plant species remains unknown.

In this study, we expanded the analysis of stored mRNA in Arabidopsis seeds to aged seeds of wheat (*Triticum aestivum*) and canola (*Brassica napus*) with the aim to address the following questions: (1) what are the *β* values for mRNA degradation in the aged seeds of wheat and canola, (2) do the overall *β* values differ between the aged seeds of these two species, and (3) how much does the overall *β* value change in the aged seeds of a species across different ageing conditions? The wheat and canola seeds were selected to represent conserved germplasm of grain and oil crop species, respectively, and both species are agriculturally important. Specifically, four sequential analyses were conducted in 2017 and 2018. The first analysis focused on the comparative seed germination kinetics of wheat or canola to Arabidopsis seeds that aged under the same acceleratedly aged (AA) conditions. The second assay was made to evaluate the possible changes of total RNA isolated in differently aged seeds from wheat, canola, and Arabidopsis. The third was to analyze the impacts of seed aging time and the mRNA length on stored mRNA degradation of both wheat and canola seeds through qPCR. The fourth was to estimate the *β* values of wheat or canola and to compare them with the reference Arabidopsis.

## 2. Results

### 2.1. Germination Kinetics for Wheat, Canola, and Arabidopsis Seeds

**Figure 1:**
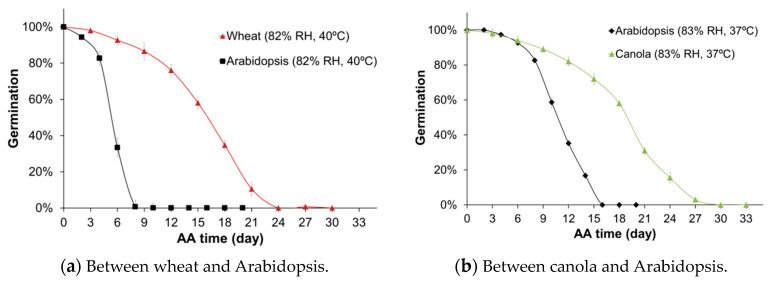
Seed germination kinetics of wheat, canola, and Arabidopsis seeds during an accelerated aging (AA) process. (**a**) Comparison between acceleratedly aged seeds of wheat and Arabidopsis under the AA conditions of 40 °C and 82% relative humidity (RH). (**b**) Comparison between acceleratedly aged seeds of canola and Arabidopsis under the AA conditions of 37 °C and 83% RH. For each AA seed lot, 50 seeds of wheat, or 100 seeds of canola, or 100 seeds of Arabidopsis were sterilized and rinsed in ddH_2_O before being plated on ½ Murashige and Skoog salts (MS) plate. Seed germination was evaluated after seven days of incubation. The germination percentages were calculated based on three replicates and the standard errors are shown.

Germination percentages of wheat seeds that were acceleratedly aged for 0, 3, 6, 9, 12, 15, 18, 21, 24, 27, and 30 days were collected and plotted against seed aging days to show germination kinetics under conditions of 40 °C and 82% relative humidity (RH). As shown in Figure 1a, seed germination percentage decreased non-linearly over aging time. For the assayed aging conditions, the change in germination was very slow for the first ten days and for the next ten days, the germination percentage decreased rapidly from above 80% to about 10%. After 24 days of AA treatment, the seeds completely lost the ability to germinate. This seed germination curve was consistent with the generalized model of seed aging, consisting of (1) an asymptomatic phase during which the ability of seeds to germinate changes relatively little, (2) a phase of “rapid mortality”, and (3) the last phase in which seeds could no longer germinate [22]. For Arabidopsis seeds that aged under the same conditions of 40 °C and 82% RH, the germination kinetics was similar to that of the AA wheat seeds, but the curve was sharper.

For canola, the change in seed germination percentage over aging time (Figure 1b) had a similar curve or trend to what was observed for wheat seeds (Figure 1a), although canola seeds were aged under 37 °C and 83% RH. The time to lose germination ability for canola seeds was relatively longer than Arabidopsis seeds that aged under the same conditions. Overall, these comparisons in germination kinetics clearly showed that wheat or canola seeds had greater aging tolerance or “lifespan” than Arabidopsis seeds under the same AA conditions.

### 2.2. Similar Quality of RNA Samples for Aged Wheat and Canola Seeds

**Figure 2:**
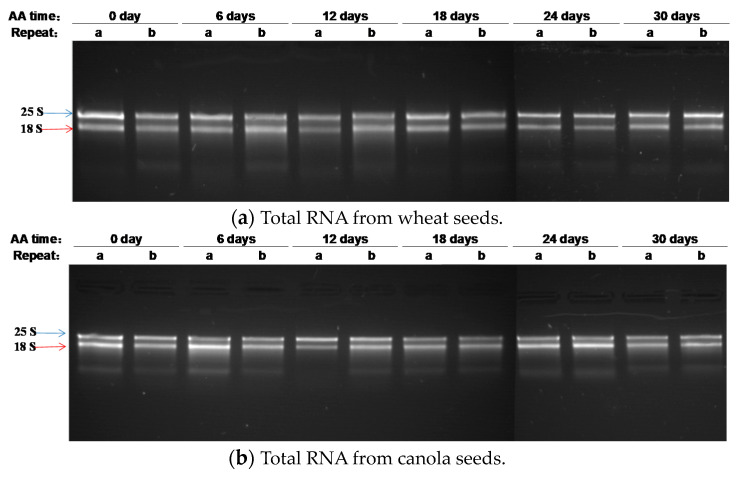
The integrity of total RNAs isolated from unaged and acceleratedly aged wheat (**a**) and canola (**b**) seeds. Total RNAs were isolated from dry seeds of wheat and canola, as shown in Figure 1 and described in the Materials and Methods. The AA days for the seeds are indicated at the top of the figure. There were six wheat or canola seed samples with two replicates of total RNAs labeled as a and b. The same amount of total RNA (0.5 μg) was loaded into each lane in the 1% agarose gel, which was subjected to electrophoresis.

Total RNAs were isolated from the AA seeds of wheat (AA for 0, 6, 12, 18, 24, and 30 days) and canola (AA for 0, 6, 12, 18, 24, and 30 days). The RNA concentration, purity, and integrity were analyzed as described in the Materials and Methods. As shown in Table 1, the concentration of total RNA varied among differently aged seeds of either wheat (122.0–444.0 ng/µL) or canola (173.0–601.0 ng/µL). However, the concentration of total RNA showed no apparent decreasing or increasing trend with the germination percentage for wheat or canola seeds. The A_260/280_ and A_260/230_ ratios reflecting RNA purity were quite similar for all the RNA samples of wheat (A_260/280_: 1.98–2.10; A_260/230_: 2.10–2.32) and canola (A_260/280_: 1.95–2.13; A_260/230_: 2.21–2.44). In addition, when 0.5 μg of the total RNA sample were loaded and run in agarose gels, there were clear 25s and 18s rRNA bands with no apparent smearing for variously aged seeds of wheat (Figure 2a) and canola (Figure 2b). Similarly, for Arabidopsis seeds that aged under 37 °C or 40 °C, the purity and integrity showed no apparent trend of decreasing or increasing compared to the unaged seeds (Table S1 and Figure S1). Such consistency in RNA purity and integrity is important for comparative analysis of stored mRNA degradation among differentially aged seeds. Thus, we selected from Table 1 all “b” replicate samples of wheat and the replicates of “a”, “a”, “a”, “a”, “b”, and “a” canola seed samples (aged for 0, 6, 12, 18, 24, and 30 days, respectively) to perform further assays of cDNA synthesis, reverse transcription PCR, and quantitative real-time PCR analyses.

### 2.3. Correlation of mRNA Degradation with Aging Time and Fragment Length

Initially, 19 candidate wheat genes and 19 candidate canola genes were randomly selected from those assayed in the companion study [45], as almost all assayed stored mRNAs showed degradation, to survey the presence of stored mRNAs in seeds of both species. For simplicity and ease of reference, the candidate genes were referred to by codes such as W1 and W2 for wheat genes (Table S2) and Bn1 and Bn2 for canola genes (Table S3). An initial survey by reverse transcription PCR (RT-PCR) was done with a fragment size of about 950 bp (starting from or near the STOP codon) used for wheat genes, while about 1500 bp fragments were used for canola genes. Note that some primers were specifically designed to start with a few base pairs apart from the STOP codon to avoid sequences with high GC contents. The results showed that under the assayed RT-PCR conditions a clear and specific cDNA band was amplified for nine of the 19 wheat genes (W2, W3, W7, W10, W11, W12, W14, W16, and W17; Figure S2a) and ten of the 19 canola genes (Bn1, Bn2, Bn3, Bn4, Bn9, Bn11, Bn12, Bn13, Bn17, and Bn18; Figure S2b). Meanwhile, five canola genes (Bn7, Bn8, Bn14, Bn16, and Bn19) displayed a weak but visible band on the agarose gel (Figure S2b).

**Figure 3:**
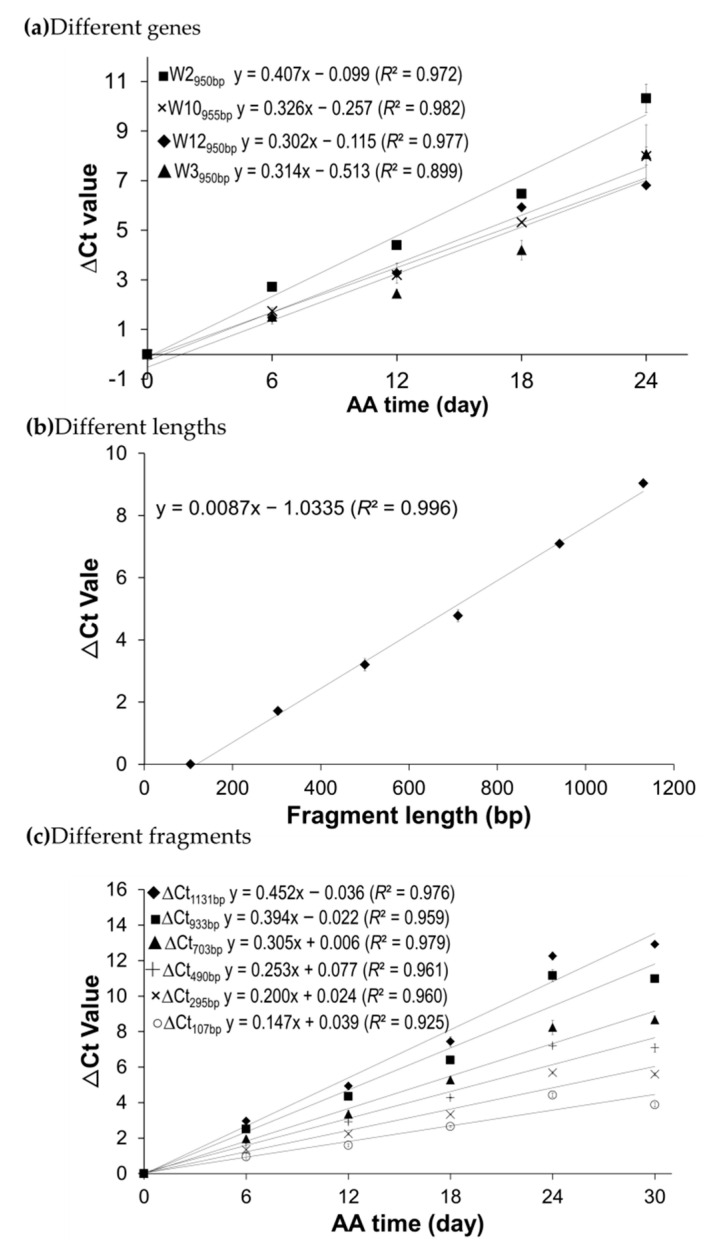
Relationships among the ΔCt value, cDNA fragment size, and seed aging time as revealed from qPCR analysis of wheat seeds. (**a**) The relationship between the ∆Ct values of stored mRNAs of four wheat mRNA genes and seed aging time. cDNAs of control and four acceleratedly aged seed samples for the indicated aging time (in days) were used. Stored mRNAs of four genes (with the fragments of W2_950bp_, W3_950bp_, W10_955bp_, and W12_950bp_) were analyzed. (**b**) Relationship between the ∆Ct value and cDNA fragment size of the wheat W2 gene. cDNAs of the control (100% germination) and 30-day AA seeds were used to obtain ∆Ct values of the fragments on the W2 mRNA; the W2 fragments are W2_107bp_, W2_295bp_, W2_490bp_, W2_703bp_, W2_933bp_, and W2_1131bp_. (**c**) Correlation of the ∆Ct value with seed aging time analyzed using six different cDNA fragment lengths of gene W2. cDNAs of the control and five AA seed samples under variable aging days were used, and the six W2 fragments in Figure 3b were used. Each ∆Ct value was obtained based on three technical replicates and its standard error was shown. The linear regressions had *p* < 0.01 in the *F*-test for each of the regressions.

**Figure 4:**
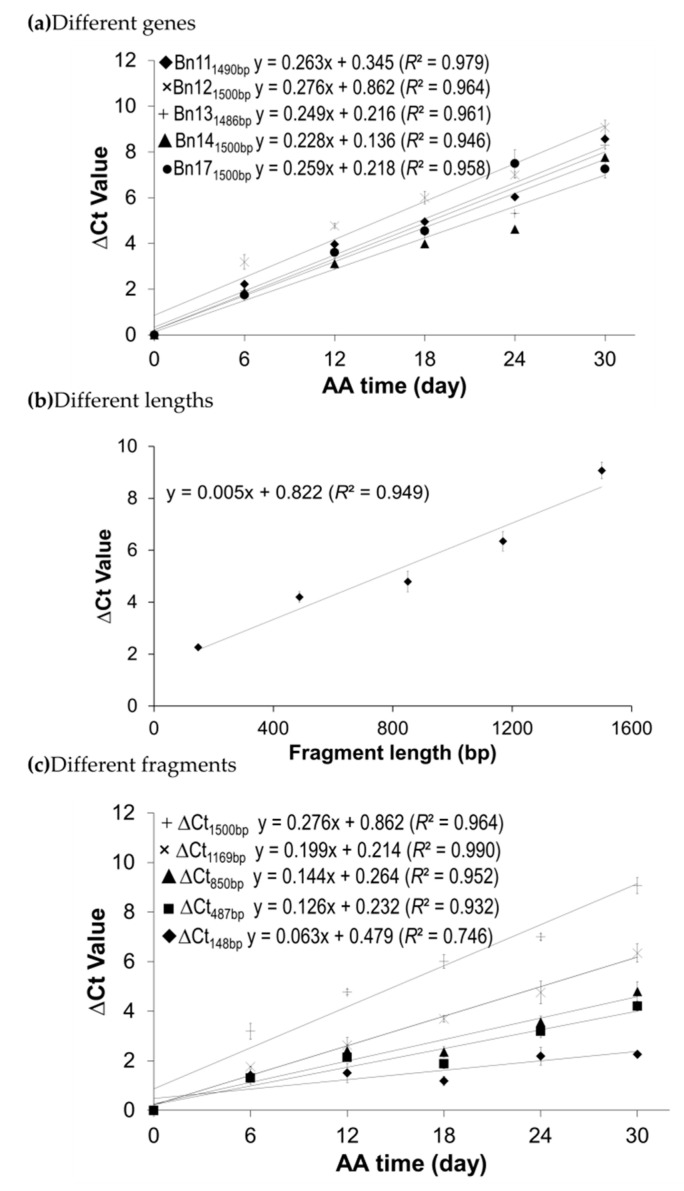
Relationships among the ∆Ct value, cDNA fragment size and seed aging time as revealed by qPCR analysis of canola seeds. (**a**) The relationship between the ∆Ct values of stored mRNAs of five canola genes and seed aging time. cDNAs of control and five acceleratedly aged seed samples for variable aging days were used. The ∆Ct values between the aged and control samples for Bn11_1490bp_, Bn12_1500bp_, Bn13_1486bp_, Bn14_1500bp_, and Bn17_1500bp_ (the fragment length analyzed is indicated by the subscript) were determined. (**b**) Relationship between the ∆Ct value and cDNA fragment size of canola gene Bn12. cDNAs of the control (100% germination) and 30-day AA seeds were used to obtain ∆Ct values of the fragments on gene B12; the fragments were Bn12_148bp_, Bn12_487bp_, Bn12_850bp_, Bn12_1169bp_, and Bn12_1500bp_. (**c**) Correlation of the ∆Ct value with seed aging time analyzed using five cDNA fragments of Bn12. cDNAs of the control and five AA seed lots under variable aging days were used; the five fragments in Figure 4b were used. The linear regressions had at least a *p* < 0.05 in the *F-*test for each of the regressions.

Based on the initial survey, only genes with relatively strong PCR amplifications of stored mRNA were selected to ensure that the selected mRNAs after degradation under lengthy AA treatments could still be detected in a qPCR assay. Thus, four wheat genes (W2, W3, W10, and W12; Figure S2a) and five canola genes (Bn11, Bn12 Bn13, Bn14, and Bn17; Figure S2b) were selected for further analyses. Note that the melt curves for the qPCR on the stored mRNAs of these two sets of genes can be found in Figure S3. Each melt curve revealed one sharp peak corresponding to the PCR product when the melting temperature rose to higher than 85 °C for each of the wheat or canola genes, indicating that the target amplicons were amplified specifically. Additionally, one minor peak was displayed at the melting temperature of ~70 °C, which should not have had a major effect on the qPCR assay. For wheat, the ∆Ct values in qPCR assay for each of the four genes showed a high correlation with aging time with a correlation coefficient near or above 0.90 (*p* < 0.01; Figure 3a). At the same time, the regression slopes were very similar among the selected fragments with similar lengths, suggesting that the assayed fragments of similar length had similar mRNA degradation rates. In the case of canola, the ∆Ct values in qPCR assays for each of the five genes also displayed a remarkably high correlation with the seed aging time with a correlation coefficient above 0.90 (*p* < 0.01; Figure 4a). The regression slopes were also similar among the five canola genes, as observed in wheat.

To study the correlation of mRNA degradation with fragment size, fragments with different lengths on one mRNA were used in qPCR analysis as performed by Zhao et al. [45]. Additionally, unaged seeds were used as the reference and seeds aged for 30 days were employed to have more mRNA degradation (and thus to generate larger ∆Ct values) for better statistical analysis. For wheat, ∆Ct values of six fragments on gene W2 (W2_107bp_, W2_295bp_, W2_490bp_, W2_703bp_, W2_933bp_, and W2_1131bp_, see Table S2 and Figure S4a) were generated by qPCR analyses. For the designations, W2_107bp_ means a 107 bp fragment of W2 mRNA. As shown in Figure 3b, there was a very high correlation between the ∆Ct value and fragment size (*R*^2^ = 0.996 and *p* < 0.0001). A similar correlation was also found on the five fragments of the canola gene Bn12 (Bn12_148bp_, Bn12_487bp_, Bn12_850bp_, Bn12_1169bp_, and Bn12_1500bp_ in Table S3 and Figure S4b) using the AA seed samples acceleratedly aged for 0 and 30 days (Figure 4b). These results indicated that the level of undamaged stored mRNAs decreased with the increasing length of the cDNA fragment analyzed, which was consistent with previous finding in Arabidopsis [45]. Interestingly, when we specifically analyzed the degradation trend of the six W2 fragments and the five Bn12 fragments with seed aging time, all of the fragments had a high correlation between ∆Ct values and AA times (Figure 3c for wheat and Figure 4c for canola).

### 2.4. Similar Rate of mRNA Degradation in Aged Seeds of Wheat, Canola and Arabidopsis

The stored mRNA degradation per nucleotide per day (or *β* value) was estimated using the ∆Ct values of the four wheat fragments (Figure 3a) and five canola fragments (Figure 4a), as described in the Materials and Methods. Such estimation assumed that any damage on an mRNA strand to be equivalent to “a break” on a nucleotide if the damage prevents the reverse transcriptase from passing through the particular nucleotide on the mRNA template in a reverse transcription reaction. After seed aging, one nucleotide could be either “a break” at the probability *β* or intact with a probability of 1- *β*. For wheat, the estimated *β* values were close to each other among different fragments or time points (Table 2), and there were no statistically significant differences in the average *β* values among different seed aging days, as revealed by the ANOVA analysis. However, the average *β* value for the gene W2 is significantly higher than those of W3, W10, and W12 in the ANOVA analysis (with all *p* < 0.0001). The overall *β* value for the four wheat genes was 2.26 ± 0.51 × 10^−4^ at 40 °C and 82% RH. The estimated *β* values for the five canola genes were also very similar, and there was no significant differences in the average *β* values among different seed AA times. However, the average *β* value for Bn12 was significantly larger than Bn14 (*p* = 0.0240), based on the ANOVA analysis (Table 3). The overall *β* value for the five canola genes was 1.37 ± 0.31 × 10^−4^ under 37 °C and 83% RH (Table 3).

**Figure 5:**
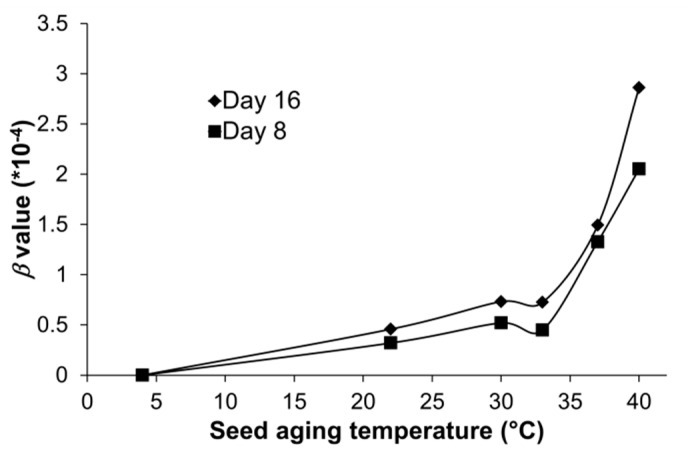
Estimated *β* values for one stored mRNA gene in Arabidopsis seeds aged at different temperatures and days. Seeds were treated at 22, 30, 33, 37, and 40 °C for 8 or 16 days, with seeds stored at 4 °C used as the unaged control. The *β* value was estimated as described in the Materials and Methods using the ∆Ct value for the 2000bp fragment of Arabidopsis gene At1g74310; this gene was previously assayed by Zhao et al. [45]. Each *β* value was obtained from three technical replicates.

The *β* values for Arabidopsis seeds aged at 37 °C or 40 °C (Figure 1) were also estimated for comparison with wheat or canola. The estimation of *β* values for Arabidopsis seeds that aged under 4 °C (with 88% RH), 22 °C (with 85% RH), 30 °C (with 84% RH), and 33 °C (with 84% RH), was made to evaluate the temperature effects on stored mRNA degradation. As shown above, the *β* value was less sensitive to seed aging time, and thus only the seeds aged for eight and 16 days were further analyzed (Figure 5). These analyses showed quantitatively that stored mRNA degradation increased speedily with increasing temperatures (Figure 5), indicating that stored mRNA degradation accelerated at the higher temperatures for the accelerated seed aging.

Further, the overall *β* values for Arabidopsis seeds aged under 37 °C or 40 °C were compared to the values for wheat or canola. Surprisingly, under the same conditions of 40 °C and 82% RH, the overall *β* value of Arabidopsis (2.46 ± 0.45 × 10^−4^ in Table 4) showed a non-significant difference (*p* = 0.3925 in the Student’s *t*-test) to the overall *β* value of wheat (2.26 ± 0.51 × 10^−4^ in Table 2). Meanwhile, under the same conditions of 37 °C and 83% RH, the overall *β* value of Arabidopsis (1.41 ± 0.13 × 10^−4^ in Table 4) did not display a significant difference (*p* = 0.7694 in the Student’s *t*-test) to the overall *β* value of canola (1.37 ± 0.31 × 10^−4^ in Table 3). These similar *β* values suggest that the basic RNA damage was similar between aged seeds of wheat (or canola) and Arabidopsis.

## 3. Discussion

### 3.1. Stored mRNAs Were Gradually Degraded at a Constant Rate over Aging Time in Wheat and Canola Seeds

We reported previously that degradation of stored mRNAs was highly correlated with seed aging in naturally and acceleratedly aged Arabidopsis seeds [45]. Using qPCR, we analyzed the changes in stored mRNAs (as reflected by the corresponding cDNAs) in terms of the fragment size and seed aging time. These results allowed us to observe two fundamental characteristics of stored mRNA degradation during Arabidopsis seed aging. First, when different fragment lengths of the same stored mRNA were compared at a given aging time point, the ∆Ct value of the qPCR increased linearly with the mRNA length, indicating that the damage or degradation of a stored mRNA occurred randomly along the length of a stored mRNA. Such degradation patterns were also observed by Fleming et al. [41]. Second, when a given length of a stored mRNA was used, the ∆Ct value was highly correlated with seed aging time, indicating that the time for the mRNA level to decrease by 50% (∆Ct = 1) is constant or, in other words, a stored mRNA degrades at a constant rate over the aging time [45].

In this study, we observed similarly very tight correlations between the ∆Ct value and seed aging time in wheat and canola seeds. Typically, the correlation coefficients had an *R*^2^ value of more than 0.95. This correlation is valid from the unaged seeds to an aged time far beyond the point at which aged seeds lose the ability to germinate completely. Results from the three different plants, as well as those of 11 plant species in two other studies [29,30], suggest that stored mRNA degradation during seed aging is a general phenomenon for seeds of many plants. The tight correlations of the ∆Ct value with mRNA fragment length and seed aging time, observed previously in Arabidopsis [45] and here in wheat and canola, strongly suggest that the damage on a stored mRNA occurred randomly along the length of a stored mRNA and accumulated evenly with seed aging time under the same conditions [41]. However, the generality of such stored mRNA degradation remains to be determined with the selection of more genes assayed across the large genomes of wheat and canola and some attention may need to be paid to specific genes associated with seed storage.

### 3.2. The β Value Can Be Used to Describe the Rate of mRNA Degradation Quantitatively in Different Plants

Based on these characteristics of stored mRNA degradation during seed aging, we have reported the development of a new parameter, the *β* value, to quantitatively describe the average frequency of “breaks” per nucleotide per day in a stored mRNA under the constant aging conditions [45]. The *β* value can be estimated based on the ∆Ct values using an equation we reported. Although it is an indirect estimate based on the level of cDNA derived from the stored mRNA, the *β* value provides a new, simple, and quantitative measurement for analyzing stored mRNA degradation and seed aging. It should allow comparisons of different mRNAs, different regions of the same mRNA, and different aging conditions in seeds of different species. The results from these experiments indicate that the *β* values were very similar for different genes of wheat or canola (Table 2 and Table 3), although significant differences among different genes could be found in the ANOVA analysis. In addition, we also determined the rate of stored mRNA degradation at different temperatures using Arabidopsis seeds. The result showed that the *β* values increased with increasing temperature (Figure 5). It is well known that plant seeds age faster at increasing temperatures [35] and it is thus interesting to see that the rate of stored mRNA degradation depends on aging temperature as well. The result (Figure 5) also underlined the need for studying the stored mRNA degradation under seed bank conditions, as seed aging in seed bank conditions of lower temperature and RH may differ from those under the assayed AA conditions.

One interesting question is whether seed stored mRNAs in different species are degraded with similar or different rates. Since the *β* values for different genes in one species are mostly similar, we could generate an overall *β* value based on several or many different genes, which can be compared among seeds of different species aged under the same conditions. The results showed, surprisingly, that the overall *β* value for wheat (2.26 ± 0.51 × 10^−4^ in Table 2) was not significantly different from the overall *β* value for Arabidopsis (2.46 ± 0.45 × 10^−4^ in Table 4) at 40 °C and 82% RH. Additionally, the overall *β* value for canola (1.37 ± 0.31 × 10^−4^ in Table 3) was not significantly different from that for Arabidopsis (1.41 ± 0.13 × 10^−4^ in Table 4) at 37 °C and 83% RH. Although only three species and limited number of genes were analyzed, the finding of similar *β* values for seeds of different plant species aged under the same conditions is remarkable, considering the apparent differences in seed morphology, genome complexity, and seed aging kinetics of different plants (Figure 1). These results suggest for the first time that stored mRNAs in the seeds of the three different plants were degraded with comparable rates under the same conditions. It will be interesting to determine how wide this similarity holds among seeds of many different plants and, more interestingly, how *β* values changes with time under seed bank conditions.

The integrity of rRNA in aged seeds has been long studied [57,58,59,60,61] and RNA integrity number (RIN) was proposed to measure RNA quality [62,63]. Recently, Fleming and her colleagues applied RIN to assess seed aging status [29,30] and showed that RNA integrity decreased during seed aging in several plant species. Our study did not measure RIN in aged wheat and canola seeds, and it is impossible to make a direct comparison of sensitivity between RIN and *β* value. However, a visualization of the RNA gels obtained in this study revealed little rRNA smearing (e.g., see Figure 2), suggesting that the variation in RIN, if measured, would be small among the assayed samples. The suggested RIN range might be similar to those ranging from eight to nine among the samples of naturally aged and AA seeds of Arabidopsis [45]. Thus, it can be reasoned that *β* value is more sensitive than RIN to measure seed aging status under AA treatment. However, it is possible that in some cases of naturally aged seeds with highly degraded RNAs under genebank conditions [4], RIN could be sensitive to detect the aging changes.

### 3.3. β Values for AA Seeds of Arabidopsis Increased Non-Linearly with High Temperatures

One unexpected and interesting finding in this study is the observation of non-linearity between the *β* values for AA seeds of Arabidopsis and the employed high temperatures (Figure 5). There seemed to be a turning point around 33 °C, after which the *β* value or the rate of stored mRNA degradation increased much faster (Figure 5). Specifically, the *β* value roughly doubled when the temperature increased by 9 degrees from 22 °C to 33 °C, while it more than doubled when the temperature increased by 3 degrees from 37 °C to 40 °C (Figure 5). It is difficult to explain this observation for seed aging under wet and warm conditions using the classic theory of the glassy matrix [4,6] based on the solid-to-fluid transition temperature (*Tg*) for seed aging under dry and cold conditions. However, it is highly possible that the turning point of the *β* values at 33 °C under 83% RH reflected the significant changes in the physical state of the seed cytoplasm, resulting in less restriction of the molecules by the glassy matrix [4,23]. If so, we can reason that the melting temperature (*Tc*) for AA seeds of Arabidopsis under 83% RH might be around 33 °C, as *Tc* is usually higher than *Tg* [9,23,64], allowing at least for a possible mechanistic explanation for the accelerated seed aging at higher temperatures. This reasoning also suggests that *β* value could be used as a tool to study the changes in the physical or biochemical environment in seeds and that the *β* value could be a marker of a sudden change in viscosity and molecular mobility of aging seeds. However, as the possibility cannot be excluded that such mRNA breakdown simply reflected the increased impact of higher temperature on the seed-stored mRNA, more research is encouraged to further analyze the effects of temperature, RH, aging time under glassy state, and the solidification of the cytoplasm on the changes of *β* value in the future.

### 3.4. Practical Implications for Plant Germplasm Conservation

The findings of mRNA degradation in wheat and canola seeds, along with those in Arabidopsis seeds, are encouraging for the search for seed viability biomarkers. First, the stored mRNAs degraded in correlation with controlled aging times of wheat and canola seeds, which implies that the correlation can be useful for characterizing seed aging phases [22], at least under controlled aging schemes. Second, since different stored mRNAs are degraded at similar rates, only a few mRNAs can be explored as the molecular markers or an internal molecular “clock” for assessing seed aging time. Third, the similar rates of mRNA degradation in the aged seeds of wheat, canola, and Arabidopsis imply that some seed aging characteristics inferred from stored mRNAs of one plant species may be applicable to those of the other plant species. This implication may carry more weight and utility for genebanks, as seed conservation is involved with thousands of plant species [5]. Thus, stored mRNAs have the potential to be developed as a useful seed aging biomarker, like those speculated by Fu et al. [2]. However, more research is needed to investigate seed aging dynamics from stored mRNAs for seeds conserved under genebank storage conditions before the development of informative biomarkers for monitoring seed viability in genebanks [2,65].

## 4. Materials and Methods

### 4.1. Comparing Seed Germination Kinetics in Wheat, Canola, and Arabidopsis Seeds

Seeds of the common wheat cultivar “Superb”, canola cultivar “Westar”, and Arabidopsis ecotype “Columbia” were used in this study. Wheat and canola seeds were acquired from Plant Gene Resources of Canada, Saskatoon Research and Development Centre (SRDC), Saskatoon, Canada, and the acquired seeds were harvested on 2 September 2015 and on 18 August 2017 from the plants grown in the SRDC greenhouse, respectively. Arabidopsis seeds were harvested on 16 June 2017 from the plants grown in one of our laboratories (Hong Wang). The assayed seeds were randomly sampled from the harvested seeds. The sampled wheat and canola seeds were sealed and stored in an airtight aluminum bag in a 4–5 °C refrigerator, while the sampled Arabidopsis seeds were sealed and stored under 4–5 °C in 2 mL microtubes with airproof screw caps.

Ideally, one should examine the dynamics of stored mRNA degradation in seeds aged under genebank storage conditions, but such materials may take years to be generated and are not available for our study. Additionally, it is difficult to have wheat and canola seeds aged naturally under constant conditions for years and seeds aged naturally under variable conditions may vary in quality, compounding the degradation analysis. Thus, we applied AA treatment in this study and produced a set of aged seeds under milder conditions than the ones used in other studies [66,67,68]. The AA treatments were performed on the sampled seeds using the conditions similar to those described previously [69]. Briefly, one thin layer of dry seeds for each species was distributed on a piece of paper which was tiled horizontally on the bottom of a mesh holder. The mesh holder was placed into a plastic container (22.0 × 24.0 × 35.0 cm) with four bottles of saturated KCl solution at the bottom to create high humidity of about 82–83% at 37 to 40 °C. The plastic container was capped with a lid, sealed with plastic wrap, and placed into an incubator. For seed aging comparison of wheat to Arabidopsis under 40 °C and 82% RH, 11 AA time points were used for wheat (0, 3, 6, 9, 12, 15, 18, 21, 24, 27, and 30 days) and Arabidopsis (0, 2, 4, 6, 8, 10, 12, 14, 16, 18, and 20 days) to achieve varying germination percentages from 100% to 0%. As the Arabidopsis seeds under 40 °C and 82% RH lost viability completely in eight days, we examined their germinations for every two days, rather than every three days, as for wheat seeds, to make the kinetic plot more smooth and informative. For the comparison between canola and Arabidopsis under 37 °C and 83% RH, a similar frame of 11 AA time points were used for canola (0, 3, 6, 9, 12, 15, 18, 21, 24, 27, and 30 days) and Arabidopsis (0, 2, 4, 6, 8, 10, 12, 14, 16, 18, and 20 days). The aged seed samples of wheat (~180 seeds), canola (~350 seeds), and Arabidopsis (~100 mg seeds) were removed from the container after treatment, air-dried for 3 days at room temperature, and then stored at 4–5 °C until use.

For seed germination tests, seeds were firstly sterilized with 20% bleach for 15 min, followed by four rinses with sterilized ddH_2_O. Then the surface-sterilized seeds were soaked with distilled water in tubes and stratified for two days at 4–5 °C in the dark. After stratification, the seeds were sown on plant culture medium in Petri plates containing ½-strength Murashige and Skoog salts [70], 1% *w/v* sucrose, and 0.7% *w/v* agar. The culture plates were incubated in a chamber with the conditions of 20 °C, 16 h/8 h photoperiod, and light intensity of 90 ± 10 μmoles m^−2^ sec^−1^ for seven days. After incubation, once the radicle for a seed was equal or longer than the length of the seed itself, it was considered as a germinated seed. For wheat and canola, 50 seeds were sown in each plate, while 100 seeds were sown in each plate for Arabidopsis. The average germination percentage for each seed sample was obtained from three replicates.

### 4.2. Total RNA Analysis for Differently Aged Seeds of Wheat, Canola, and Arabidopsis

Total RNAs were extracted from dry seeds of wheat aged for 0, 6, 12, 18, 24, and 30 days and canola aged for 0, 6, 12, 18, 24, and 30 days to analyze stored mRNA degradation with time. To conduct a preliminary exploration on the stored mRNA degradation under different temperatures, Arabidopsis seeds aged under 4 °C, 22 °C, 30 °C, 33 °C, 37 °C, and 40 °C for eight days and 16 days were also extracted for total RNAs. The extraction was done following a modified protocol as described by Onate-Sanchez and Vicente-Carbajosa [71]. Briefly, five wheat seeds, ten canola seeds, or 50 mg Arabidopsis seeds were frozen with liquid nitrogen in a mortar and were ground into powder with a pestle. The powder was well suspended with a mixture of 550 μL buffer (0.4 M LiCl, 0.2 M Tris (pH 8.0), 25 mM EDTA, 1% SDS) and 550 μL chloroform in a 1.5 mL tube. Following centrifugation for 3 min, 500 μL of the upper phase were pipetted out and mixed with 500 μL phenol in a new 1.5 mL tube. After re-suspension and 5 min incubation, the solution was mixed with 200 μL chloroform thoroughly. The mixture was centrifuged for 3 min and 500 μL upper phase were pipetted out and mixed with 170 μL 8 M LiCl in a new 1.5 mL tube. After incubation in a −20 °C freezer for 30 min, the mixture was centrifuged for 15 min in a 4 °C centrifuge. After discarding the supernatant, the pellet was digested with DNase (Roche Holding AG., Basel, Switzerland) for 20 min. Then a mixture containing 7 μL 3 M NaAc, 250 μL 100% ethanol, and 500 μL DEPC-H_2_O was added into the DNase-digested mixture. After 10 min centrifugation at 4 °C, the supernatant was pipetted out and mixed with a solution containing 43 μL 3 M NaAc and 750 μL 100% ethanol, followed by 30 min incubation at −20 °C and 10 min centrifugation at 4 °C. The supernatant was discarded and the pellet was washed with 70% ethanol two times and air-dried for 10 min. Lastly, the pellet of total RNA was dissolved in 20 μL DEPC H_2_O. In this method, 22,000× *g* was used for all the centrifugations. The RNA samples were stored at −80 °C until use.

The quantity and purity for total RNA were measured using a NanoDrop 8000 spectrophotometer (Thermo Fisher Scientific Inc., Waltham, MA USA). The readings of A_260/280_, A_260/230_, and concentration were obtained for each sample. A pure RNA sample should have a reading of A_260/280_ from 1.8 to 2.0, and a reading of A_260/230_ from 2.0 to 2.2. Total RNA integrity was also roughly evaluated by 1% agarose gel electrophoresis. In the gel electrophoresis analysis, the relative brightness of 25S and 18S rRNA bands and the band smearing were considered as the major factors. For high-quality total RNA, the brightness of 25S rRNA should be roughly twice that of 18S, while the bands should not show a smearing.

### 4.3. qPCR Analysis of mRNA Degradation in Aged Seeds

The first-strand cDNA was synthesized in 10 μL using 0.5 μg of total RNA and an oligo (dT)20 primer with a ThermoScript RT-PCR system according to the manufacturer’s manual (Thermo Fisher Scientific Inc., Waltham, MA, USA), as shown in Figure S4. The resulting cDNA sample was added to two volumes of sterile ddH_2_O. Genes used for surveying the presence of stored mRNAs by RT-PCR are listed in Table S2 (for wheat) and Table S3 (for canola). Specifically, these genes were randomly selected from those assayed in our companion study [45], which showed that almost all assayed genes displayed similar degradation. Our companion study [45] also showed that all of the six characterized Arabidopsis genes degraded similarly in rate and thus only one (At1g74310) of these Arabidopsis genes was selected for this study. PCR products were separated by electrophoresis in 1% agarose gels and stained with ethidium bromide. The gel images were obtained with a BioDoc-It imaging system (SOMATCO, Riyadh, Saudi Arabia).

For qPCR, the cDNA as synthesized above was diluted in 1/3 with sterile water. A qPCR reaction was performed in 20 μL, which contained 10 μL 2X Green-2-Go qPCR Mastermix (Bio Basic Inc., Markham, Canada), 1.0 μL diluted cDNA, and 1.0 μL each of the two primers (to a final concentration of 0.25 μM). The genes and primers in the qPCR analysis are listed in Table S2 for wheat, Table S3 for canola, and Table S4 for Arabidopsis. Reaction conditions were as follows: 94 °C for 5 min followed by 40 cycles at 94 °C for 45 s, 60 °C for 45 s, 72 °C for 90 s, and a final extension at 72 °C for 5 min. Reactions were conducted with the CFX96 Real-Time system (Bio-Rad Laboratories, Inc., Hercules, CA, USA) and a threshold cycle (Ct) value was generated for each reaction with CFX Maestro Software (Version 3.0; Bio-Rad Laboratories, Inc., Hercules, CA, USA). For the stored mRNA of each gene, the difference in the threshold cycle (Ct) value (or ∆Ct value) between the aged and unaged seed sample was calculated from the Ct value of the aged sample minus the Ct value of the unaged control. For example, the ∆Ct value of the 30-day AA seeds was obtained by extracting the Ct value of day 0 from that of day 30, after both Ct values were separately generated from qPCR. Linear regression analysis of ∆Ct values over aging time points was made with RStudio (Integrated Development for R. RStudio Inc., Boston, MA, USA) and the Student’s *t*-test was performed with Excel 2010 to evaluate the significance level (or *P*) and the variance explained (or adjusted *R*^2^ values).

### 4.4. Estimating the Rate of Stored mRNA Degradation in Aged Seeds

The rate of stored mRNA degradation was measured by the *β* value based on the ∆Ct value generated from qPCR analysis, as developed by Zhao et al. [45]. For an mRNA script with “*n*” nucleotides, the probability of having “*x*” broken nucleotide(s) could be described based on the binomial distribution below:

$$P(x)\;=\;C_{x}^{n}{\left(t\beta \right)}^{x}{\left(1-t\beta \right)}^{n-x}$$

where *n* is the nucleotide count of the mRNA script, *t* is the aging days, and *β* is the probability that a break occurs for one nucleotide in the aging days *t*. *P*(0) is assumed as the probability that no nucleotide is broken, and we have:

*P*(0) = (1 − *tβ*)*^n^* (1)

Since the length of an mRNA template (or *n*) is usually long (or up to thousands of base pairs), Equation (1) can be approximated as:

*P*(0) ≈ *e*^−*tβn*^ (2)

As *P*(0) also reflects the proportion of the mRNA templates that were successfully reverse transcribed and amplified at the given aging days (*t*), we can establish the following approximate estimation of *P*(0):

*P*(0) ≈ *e*^-*tβn*^ ≈ 1/2^∆Ct^ (3)

Thus, the probability for a nucleotide to break for an mRNA template, consisting of *n* nucleotides and aged for *t* days (or *β* value), can be estimated via qPCR as:

*β* = ln(2^∆Ct^)/*tn* (4)

An ANOVA analysis of *β* values for different genes or different periods of aging for each crop was performed in RStudio with a custom R script (Version 3.6.0; R Foundation for Statistical Computing, Vienna, Austria). The Student’s *t*-test was performed with Excel 2010 to compare *β* values between wheat (or canola) and the reference Arabidopsis.

## 5. Conclusions

These sequential analyses revealed several major findings on stored mRNAs of the aged seeds of wheat and canola. First, the degradation of stored mRNA correlated well with controlled aging days of wheat and canola seeds. Second, mRNA degradation rates were similar among mRNAs and constant over seed aging days in either wheat or canola seeds. Third, the overall mRNA degradation rates in the aged seeds of wheat and canola were similar to those in the aged seeds of Arabidopsis under the same aging conditions. These results are significant for understanding stored mRNA degradations in the aging seeds of different species. More research of this nature will yield more insight into controlled seed aging for different plant species and the effective search for seed viability biomarkers.

## Figures and Tables

**Table 1:** Extracted total RNAs from the unaged and acceleratedly aged (AA) seed samples of wheat and canola seeds.

AA Day		Wheat						Canola					
		0	6	12	18	24	30	0	6	12	18	24	30
Germination Percentage		100.0	92.7	76.0	34.7	0.0	0.0	100.0	94.0	89.0	58.0	15.5	0.0
Yield (ng/μL)	a	228.0	144.0	444.0	212.1	122.0	217.9	363.0	390.0	374.0	334.7	173.0	593.0
	b	347.0	551.0	338.0	296.3	335.2	276.3	354.0	415.0	478.6	347.6	238.0	601.0
A_280/260_	a	2.02	2.02	2.03	2.07	2.05	2.10	2.10	2.11	2.04	2.07	1.95	2.10
	b	1.98	1.98	2.06	2.01	2.09	2.08	2.09	2.11	2.07	2.10	2.03	2.13
A_280/230_	a	2.27	2.24	2.23	2.21	2.13	2.20	2.40	2.31	2.23	2.31	2.44	2.21
	b	2.30	2.32	2.24	2.23	2.25	2.10	2.38	2.32	2.32	2.37	2.39	2.34

Note: Total RNAs were isolated from unaged and variously aged seeds of wheat and canola as described in the Materials and Methods. The AA day and seed germination percentage were indicated for each seed sample. Two biological replicates of RNA extraction were made for each seed sample, labeled as “a” and “b”. For each RNA sample, the RNA yield and purity (A_260/280_ and A_260/230_) were assessed using a NanoDrop 8000 spectrophotometer.

**Table 2:** Estimated *β* values for four wheat mRNA fragments of similar length in the AA seeds of different aging times.

Fragment	*β* Value (Break per Nucleotide per Day × 10^−4^)					
	Day 6	Day 12	Day 18	Day 24	Average	Overall
W2_950bp_	3.30 ± 0.10	2.68 ± 0.04	2.62 ± 0.06	3.14 ± 0.18	2.94 ± 0.15	2.26 ± 0.51
W3_950bp_	1.86 ± 0.22	1.49 ± 0.11	1.71 ± 0.16	2.46 ± 0.36	1.88 ± 0.18	
W10_955bp_	2.12 ± 0.15	1.94 ± 0.03	2.15 ± 0.02	2.42 ± 0.11	2.16 ± 0.09	
W12_950bp_	1.80 ± 0.31	1.99 ± 0.25	2.41 ± 0.06	2.07 ± 0.02	2.07 ± 0.11	
Average	2.27 ± 0.30	2.03 ± 0.21	2.22 ± 0.17	2.52 ± 0.19		

Note: The ∆Ct values between the control and aged seeds, as shown in Figure 3a, were used to calculate the *β* values using Equation (4) in the Materials and Methods. The *β* value was estimated from each of three technical replicates and its standard error was shown. W2_950bp_, for example, means that a 950 bp fragment of W2 mRNA (Table S2) was analyzed.

**Table 3:** Estimated *β* values for five canola mRNA fragments of similar length in the AA seeds of different aging times.

Fragment	*β* Value (Break per Nucleotide per Day × 10^−4^)						
	Day 6	Day 12	Day 18	Day 24	Day 30	Average	Overall
Bn11_1490bp_	1.73 ± 0.04	1.54 ± 0.06	1.28 ± 0.04	1.17 ± 0.04	1.33 ± 0.06	1.41 ± 0.05	1.37 ± 0.31
Bn12_1500bp_	2.46 ± 0.24	1.84 ± 0.04	1.54 ± 0.07	1.35 ± 0.03	1.40 ± 0.05	1.72 ± 0.09	
Bn13_1486bp_	1.51 ± 0.05	1.44 ± 0.02	1.15 ± 0.02	1.03 ± 0.00	1.29 ± 0.02	1.28 ± 0.02	
Bn14_1500bp_	1.44 ± 0.10	1.20 ± 0.10	1.02 ± 0.02	0.89 ± 0.02	1.20 ± 0.02	1.15 ± 0.05	
Bn17_1500bp_	1.35 ± 0.20	1.39 ± 0.01	1.17 ± 0.03	1.44 ± 0.01	1.12 ± 0.06	1.29 ± 0.06	
Average	1.70 ± 0.13	1.48 ± 0.05	1.23 ± 0.04	1.18 ± 0.02	1.27 ± 0.04		

Note: The ∆Ct values between the control and aged seeds, as shown in Figure 4a, were used to calculate the *β* values using Equation (4) in the Materials and Methods. The *β* value was estimated for each of three technical replicates and its standard error was shown. Bn11_1490bp_, for example, means that a 1490 bp fragment of Bn11 was analyzed.

**Table 4:** Estimated *β* values for one mRNA fragment in the Arabidopsis seeds under different AA temperatures and durations.

Gene	AA Days	*β* Value (Break per Nucleotide per Day × 10^−4^)				
		22 °C	30 °C	33 °C	37 °C	40 °C
At1G74310_2000bp_	8	0.32 ± 0.06	0.61 ± 0.07	0.37 ± 0.05	1.33 ± 0.11	2.05 ± 0.15
	16	0.46 ± 0.09	0.73 ± 0.06	0.73 ± 0.03	1.49 ± 0.08	2.86 ± 0.24
Overall		0.39 ± 0.10	0.67 ± 0.09	0.55 ± 0.18	1.41 ± 0.13	2.46 ± 0.45

Note: the *β* values were estimated using Equation (4) in the Materials and Methods from the ∆Ct values between the control and aged seeds. The *β* value was estimated for each of three technical replicates and its standard error was shown.

## Supplementary Materials

The following are available online at https://www.mdpi.com/2223-7747/9/12/1707/s1, Figure S1: The integrity of total RNAs isolated from Arabidopsis seeds that were acceleratedly aged under different temperatures, Figure S2: Results of candidate genes to survey the presence of stored mRNAs of candidate genes in wheat and canola dry seeds ((**a**) wheat genes; (**b**) canola genes), Figure S3: Melt curves of candidate genes used for qPCR analysis ((**a**–**d**) four wheat genes, (**e**–**i**) five canola genes), Figure S4: An illustration of amplifying fragments of different lengths from cDNA synthesized using total RNA and an oligo (dT)20 primer. Table S1: Extracted total RNAs from Arabidopsis seeds aged under different AA treatments, Table S2: List of genes (or fragments) and primers used to analyze mRNA degradation in wheat seeds, Table S3: List of canola genes (or fragments) and primers used to study stored mRNA degradation during seed aging, Table S4: The gene and primers used in qPCR analysis of changes in the stored mRNA of aged Arabidopsis seeds.
