# Supplementary material for: Analysis of Stored mRNA Degradation in Acceleratedly Aged Seeds of Wheat and Canola in Comparison to Arabidopsis
Source: Plants (Basel). 2020 Dec 4;9(12):1707. doi: 10.3390/plants9121707 (PMC7761881; doi:10.3390/plants9121707)
Supplement: Supplementary file 1 [file plants-09-01707-s001.pdf]

*Supplemental Materials for*

## Liang Zhao, Hong Wang and Yong-Bi Fu. 2020. Analysis of stored mRNA degradation in acceleratedly aged seeds of wheat and canola in comparison to Arabidopsis. Plants 20, e

**Table S1.** Extracted total RNAs from Arabidopsis seeds aged under different AA temperatures.

| AA day | | 8 days | | | | | | 16 days | | | | | |
|---|---|---|---|---|---|---|---|---|---|---|---|---|---|
| AA temperatures | | 4°C | 22°C | 30°C | 33°C | 37°C | 40°C | 4°C | 22°C | 30°C | 33°C | 37°C | 40°C |
| Yield | a | 357 | 420 | 415 | 355 | 346 | 367 | 324 | 344 | 208 | 324 | 387 | 331 |
| (ng/μl) | b | 342 | 320 | 408 | 298 | 501 | 389 | 177 | 325 | 277 | 304 | 197 | 325 |
| $A_{280/260}$ | a | 2.16 | 2.07 | 2.11 | 2.06 | 2.13 | 2.05 | 2.04 | 2.11 | 2.09 | 2.12 | 2.11 | 2.12 |
| | b | 2.15 | 2.12 | 2.10 | 2.13 | 2.10 | 2.13 | 2.09 | 2.13 | 2.10 | 2.12 | 2.15 | 2.09 |
| $A_{280/230}$ | a | 2.21 | 2.11 | 2.20 | 2.06 | 2.10 | 2.19 | 1.80 | 2.19 | 1.96 | 2.15 | 2.15 | 2.12 |
| | b | 2.14 | 2.12 | 2.12 | 2.17 | 2.19 | 2.06 | 2.07 | 2.14 | 2.00 | 2.13 | 2.17 | 2.08 |

Note: Total RNAs were isolated from unaged and aged seeds of Arabidopsis under various temperatures as described in the Materials and Methods. The AA day and temperature were indicated for each seed sample. Two biological replicates of RNA extraction were made for each seed sample, labeled as "a" and "b". For each RNA sample, the RNA yield and purity (A260/280 and A260/230) were assessed using a NanoDrop 8000 spectrophotometer.

**Table S2.** List of genes (or fragments) and primers used to analyze mRNA degradation in wheat seeds.

| Gene code | Gene name | Coding Sequence Length | Fragment analyzed (bps) | Forward primer | Reverse primer |
|---|---|---|---|---|---|
| W1 | TraesCS7A02G070100 | 1815 | 950 | TCAGGGAGCGGCGACGTTC | GCTACGACAAGCCGGTGGA |
| W2 | TraesCS5D02G425100 | 1519 | 950 | AAGTTGAACAAGTATGGTCGTC | GTATCTACCGGCTCGAACTC |
| W3 | TraesCS2B02G309900 | 2785 | 950 | CCTAGTGATGGAGTATTGTCC | CCACTAGAAGAGCTCGAACTC |
| W4 | TraesCS4A02G296000 | 1767 | 949 | GGGACTCACGGACGCAGAC | GGCGTTGATCTCGAAGCAC |
| W5 | TraesCS2A02G027800 | 2274 | 951 | CTCAGTTCATGTTCCCTGGT | TCACCACGTTGAGAAATGTCT |
| W6 | TraesCS3A02G471900 | 2282 | 951 | CGCTCGTCTGGTCCACGT | CGCTTCTTCTCCATGTCGAA |
| W7 | TraesCS2B02G567600 | 678 | 500 | CCTGCACCACCAGAAGCAC | TCCAGTTCACCACCTTCAG |
| W8 | TraesCS1A02G045700 | 1479 | 950 | CCATGCTCACCCTCCCGT | CGGCGGGAGGGCTTACGG |
| W9 | TraesCS5B02G267900 | 2418 | 950 | TCTGCAAAGGAGATTGATGAG | TTCATCTTCTTTGGACTACGTC |
| W10 | TraesCS5B02G106300 | 2460 | 955 | CTTCAAGAGAGTCGAATTGTGC | CAAGCGGCTCAGGTTCAGAGC |
| W11 | TraesCS2D02G289100 | 2832 | 900 | GCTGTGAAGAGATCACGCAC | TCAAAGCGTTGGTGTTGGATC |
| W12 | TraesCS4A02G143200 | 2910 | 950 | ATTATGCATTGAACCGAGGAGC | CTGGTCCTGGGTTGTCCTTC |
| W13 | TraesCS7A02G517700 | 2070 | 2000 | CCGTTTTCGCGGTCCTGATC | CTAAAGATACTTAAAGAGGATGC |
| W14 | TraesCS2B02G521600 | 1458 | 900 | GAGTTCAAGATCGTCCTCACC | GGATGCTAATGTAGTCAGACTG |
| W15 | TraesCS7B02G068100 | 1605 | 948 | GGCCTTATCTTTGACAAGAAGG | TTACCTTACAGTGACTATATCAT |
| W16 | TraesCS4A02G100500 | 1128 | 950 | GGTACATCTCCGCGGCGC | TGAACGAGCAATCTTCGCTGC |
| W17 | TraesCS3B02G311900 | 1899 | 950 | GACACTGCTGTAGAAGAGGC | TGCTCCGGTCTCTCGTTCC |
| W18 | TraesCS3D02G321500 | 741 | 655 | GCCTAGAGCGGCGGAGAAG | GGACAGATCAACGACCGACG |
| W19 | TraesCS3A02G277700 | 2304 | 951 | GATGGAGATGACCATTAAATAC | CTTGTCCTTGTCATCCGCAC |
| W2 | TraesCS5D02G425100 | 1519 | 107 | ATCTTGCTCGCGAAGGTAATG | GTATCTACCGGCTCGAACTC |
| | | | 295 | TGTTATACCGGTAGCTTCAGG | |
| | | | 490 | GGTATGCATTTCCGTGTATTAG | |
| | | | 703 | GCATTACTTGAATGCGACTGC | |
| | | | 933 | AAGTTGAACAAGTATGGTCGTC | |
| | | | 1131 | AGCCAATGGATCTGTTATGTAG | |

Note: A total of 19 wheat genes were tested, and each was coded with W and numbering. W2 was used to analyze the correlation between the ΔCt value and fragment size, with the following fragments of $W2_{107bp}$, $W2_{295bp}$, $W2_{490bp}$, $W2_{703bp}$, $W2_{933bp}$, and $W2_{1131bp}$. Four genes with similar fragment lengths $W2_{950bp}$, $W3_{950bp}$, $W10_{955bp}$, and $W12_{950bp}$ were assayed.

**Table S3.** List of canola genes (or fragments) and primers used to study stored mRNA degradation during seed aging.

| Gene code | Gene name | Coding sequence length (bps) | Fragment analyzed (bps) | Forward primer | Reverse primer |
|---|---|---|---|---|---|
| Bn1 | BnaA01g34230D | 1659 | 1001 | GGTCATAATAAGGTTTACAAGTC | TAGGTATCATATGAACAAGCTTG |
| Bn2 | BnaC04g38000D | 1080 | 1001 | GGTCATAATAAGGTTTACAAGTC | TAGGTATCATATGAACAAGCTTG |
| Bn3 | BnaC06g14690D | 1062 | 1000 | CTGTAACTGGATAACTAGTACTG | AGATGGAGCCTCAACAGCAG |
| Bn4 | BnaC05g37990D | 1470 | 1004 | CGATCTGCACTATGATCCTTAC | GATTACGTGGTACAATTGCATC |
| Bn5 | BnaA08g15530D | 2301 | 1000 | GAGTGCTTCTGCTTGATGGC | CAGTTGGTTAATATCTCAGCTC |
| Bn6 | BnaC07g44190D | 3145 | 1000 | GTGCTGCAGAAGCGGAGAG | CATTCCAGAACCTCAAAGTCTC |
| Bn7 | BnaC08g42010D | 1493 | 1000 | GAGATGATCCTTCCCATTCAC | CTTCAGCCATAATCCTCCTTG |
| Bn8 | BnaC09g16410D | 1443 | 928 | GGTATAAGTTTATATGGTTCGAAC | TGGATTCGACCTTGTCACTTG |
| Bn9 | BnaCnng08950D | 1453 | 995 | TATTCGTGGGTGGGAAAGGAG | TCAACTCTTGATCCGTTCCAG |
| Bn10 | BnaA02g02620D | 1965 | 1938 | GGTGTATGGAGAATCTGCTGG | CAGCTGCTTATGCAACTCTTC |
| Bn11 | BnaA06g10730D | 1490 | 1490 | ATGACTCTCAGAGACAGGCC | CAACCTCTTCTATCTTCGGAC |
| Bn12 | BnaA09g55830D | 2925 | 1851 | AGCAGTACTGAAGGAAGTCAC | TCGATAGGAGCATTGTTCTCG |
| Bn13 | BnaAnng00830D | 1681 | 1486 | CGTTACAACTCTGGTGCTGTC | AGAGCCTTGACGCATTGATTG |
| Bn14 | BnaC01g16200D | 2139 | 1500 | ACCAAGGCTGTTATCACTGTG | GTCGATCACATCATCTCCATC |
| Bn15 | BnaC01g39690D | 2415 | 1920 | CTATGCAGACAAGGAGAATGG | TCCACTTCAACTGGTTCAACG |
| Bn16 | BnaC03g03860D | 2046 | 1946 | ATCACTGAACGGTCAGAACTG | TCTTCACTTCCTCGTAGTCAG |
| Bn17 | BnaC03g13930D | 1731 | 1731 | AACAACACCTTGACCATCGTC | CATCTTGCTACCTTCAGCATC |
| Bn18 | BnaC04g12620D | 1692 | 1690 | TGGCAGAACCAGCATACACAG | CACTTCCTCTGAAGAGTAACC |
| Bn19 | BnaC06g23110D | 2652 | 1800 | CTATGTTAGCTAGAGGTCAGC | CCTCAATCCTCATCTTCTTCAC |
| | | | 148 | AGAGAGTGATCCAACAGATGG | |
| | | | 487 | ACTGTGTTGTGATTATGACCTC | |
| Bn12 | BnaA09g55830D | 2925 | 850 | GTCTCTCTGATCCTAACCGTC | GTTGCTTGTCTGGTCAACATC |
| | | | 1169 | TCTGCTGAACGTGAATACGAC | |
| | | | 1500 | TACATCACTGAACGCTTCCTG | |
| Bn17 | BnaC03g13930D | 1731 | 1500 | AACAACACCTTGACCATCGTC | CATCTTGCTACCTTCAGCATC |

Note: A total of 19 Canola genes were used and each was coded with Bn and numbering. Five fragments on Bn12 ($Bn12_{148bp}$, $Bn12_{487bp}$, $Bn12_{850bp}$, $Bn12_{1169bp}$, and $Bn12_{1500bp}$) were used to analyze the correlations between the ΔCt values and mRNA fragment sizes. For the comparisons of different genes with similar fragment lengths, fragments $Bn11_{1490bp}$, $Bn12_{1500bp}$, $Bn13_{1486bp}$, $Bn14_{1500bp}$, and $Bn17_{1500bp}$ were used.

**Table S4.** The gene and primers used in qPCR analysis of changes in the stored mRNA of aged Arabidopsis seeds.

| Gene name | Coding sequence length (bps) | Sequence length (bps) | Forward primer | Reverse primer |
|---|---|---|---|---|
| At1g74310 | 2736 | 2000 | TAGTTGCTGGTGCTAAATACC | TTAATCCTCGATCATTTCCTCA |

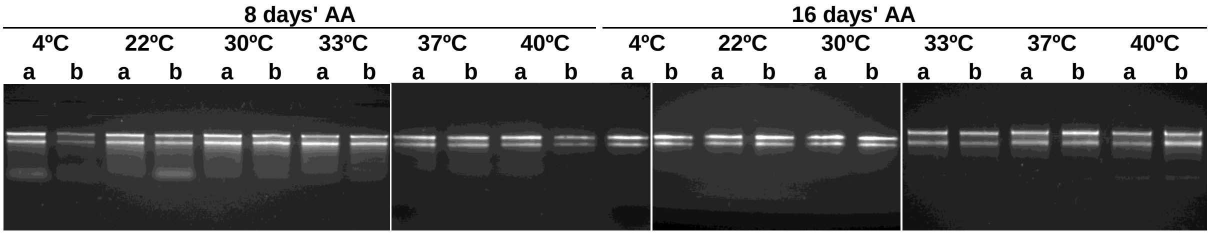


**Figure S1.** The integrity of total RNAs isolated from Arabidopsis seeds that acceleratedly aged under different temperatures. Total RNAs were isolated from dry seeds of Arabidopsis using the modified protocol as described in the Materials and Methods. The AA temperatures for the seeds were indicated on the top of the rRNA bands; there were six temperatures with two biological replicates of total RNAs, labeled as "a" and "b". The same amount of total RNA (0.5 μg) was loaded into each lane and subjected to electrophoresis.

**a:** Wheat genes

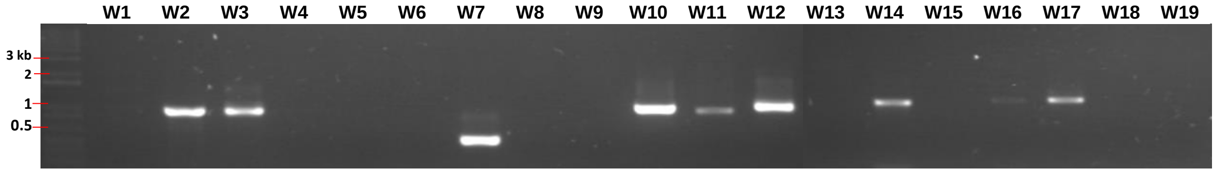


**b:** Canola genes

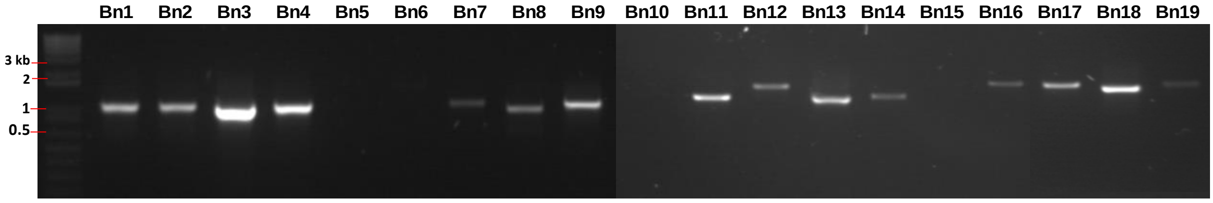


**Figure S2.** Presence of stored mRNAs of candidate genes in wheat and canola dry seeds. **a:** Nineteen wheat genes (as listed in Table S2) were used to determine the presence of stored mRNAs by RT-PCR. **b:** Nineteen canola genes (as listed in Table S3) were used to determine the presence of stored mRNAs by RT-PCR. cDNA reverse-transcribed from total RNA of unaged dry seeds was used in RT-PCR with primers specific for each of the genes. The PCR products were subjected to electrophoresis in 1% agarose gel.

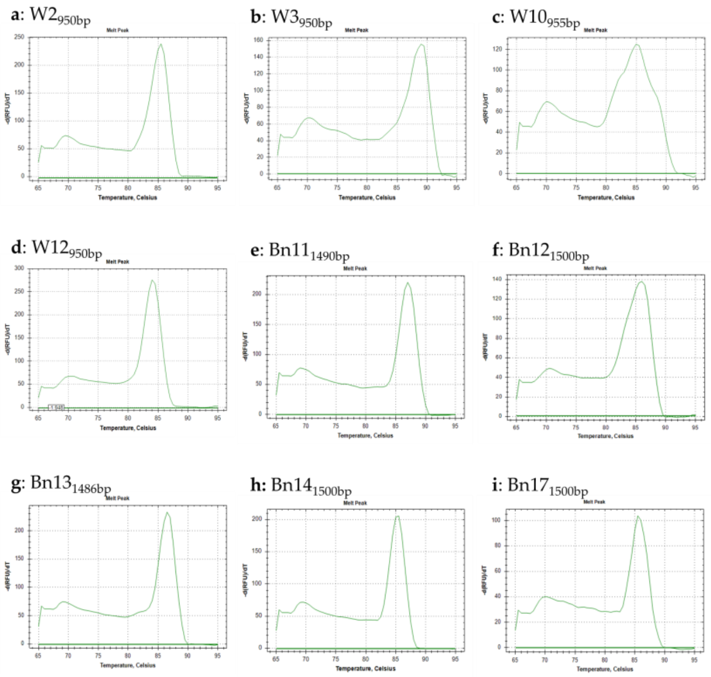


**Figure S3.** Melt-curves of candidate genes used for qPCR analysis. Figures **a-d** are melt-curves for the four wheat genes of W2$_{950bp}$, W3$_{950bp}$, W10$_{955bp}$ and W12$_{950bp}$, while figures **e-i** are melt-curves for the five canola genes of Bn11$_{1490bp}$, Bn12$_{1500bp}$, Bn13$_{1486bp}$, Bn14$_{1500bp}$, and Bn17$_{1500bp}$.

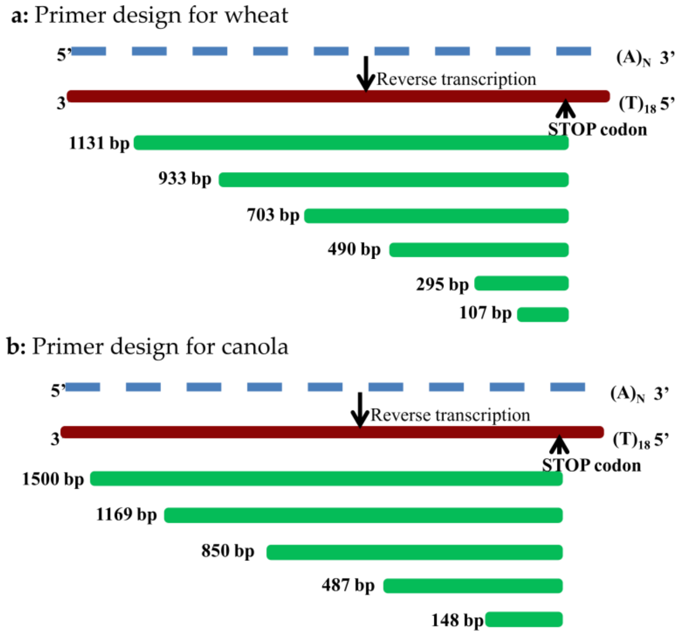


**Figure S4.** An illustration of amplifying fragments of different lengths from cDNA synthesized using total RNA and an oligo (dT)20 primer. The first-strand cDNA was firstly synthesized from total RNAs with an oligo (dT)20 primer. Then fragments of different lengths were amplified separately using the same reverse primer but different forward primers in PCR reactions.
